# Monitoring medication response in ADHD: what can continuous performance tests tell us?

**DOI:** 10.1007/s00406-021-01319-y

**Published:** 2021-08-21

**Authors:** K. Cedergren, S. Östlund, J. Åsberg Johnels, E. Billstedt, M. Johnson

**Affiliations:** Gillberg Neuropsychiatry Centre, Sahlgrenska Academy, University of Gothenburg, Kungsgatan 12A, 411 19 Gothenburg, Sweden

**Keywords:** ADHD, Children, Adolescents, Long-term medication, Effectiveness, Continuous performance test

## Abstract

Documenting effectiveness of ADHD medication is essential throughout the course of treatment. A rating scale and a continuous performance test (CPT) with motion tracking were used to study the effect of ADHD medication including compliance during one year. Children (*N* = 78, age 6–18 years) with ADHD were tested with the QbTest at baseline, visit 1 (1 month after baseline) and visit 5 (12 months after baseline). The ADHD-Rating scale was rated by investigator interview at the same visits. QbTest results and ADHD-RS ratings showed reductions in symptoms on all cardinal parameters of the QbTest and on all ADHD-RS subscales between baseline and 1 month and between baseline and 12 months. There was a weak but significant correlation between the total change scores on the two measures from baseline to 1 month. Eighteen participants dropped out of the study before visit 5; at baseline, these children showed significantly lower results on the inattention parameter of the QbTest, with faster reaction time and lower variation in reaction time, suggesting they suffered less problems with inattention. Both the QbTest and the ADHD-RS showed robust ADHD symptom improvements indicative of medication effect, and the QbTest results might also predict non-compliance of medication. Further research is warranted to increase knowledge about reliable monitoring of long-term medication and compliance.

## Introduction

Attention-deficit/hyperactivity disorder (ADHD) is characterized by a persistent pattern of symptoms of inattention and/or impulsivity/hyperactivity that clearly affects development and level of functioning in two or more life domains, such as in school, at home, with friends or in leisure activities [1]. Symptoms typically emerge during childhood and diagnostic criteria require that some symptoms have been present before the age of 12. However, very often difficulties associated with ADHD become even more apparent in later school years when higher levels of self-directed attention and executive functioning are required to succeed in school [2]. These difficulties, and failure to achieve, continue to affect occupational, academic and daily level of functioning even in adult life [3] and contribute to the elevated risk of developing psychiatric problems.

The community prevalence of ADHD among school aged children is estimated to be between 2 and 7% with an average of about 5% worldwide [3, 4]. Some recent studies show an even higher prevalence [5, 6], making it one of the most common neurodevelopmental disorders. In Sweden, as in many other European countries and in North America, an increase in public awareness of the disorder has resulted in a substantial rise in demand for access to services for diagnostic assessment as well as medical and psychosocial follow-up [3]. In addition, early identification and adequate intervention have been found to potentially alter developmental trajectories and prevent negative outcome [7]. Thus, the task of providing time-efficient care without compromising quality is indeed an important challenge for every psychiatric health professional, making brief, easily administered and objective tests for assessment and follow-up attractive.

The positive short-term effects of ADHD medication on core ADHD symptoms are well documented [8]. One recent Swedish register study reported that children with ADHD, taking medication for three months, improved their academic achievement with outcome measures such as eligibility to upper secondary school and higher-grade point averages [7]. Monitoring medical treatment in ADHD has traditionally relied on self, parent, teacher and/or clinician ratings. Although ecological validity of rating scales, such as the Attention-deficit/hyperactivity disorder rating scale (ADHD-RS), are relatively high in controlled studies [9], using them as a measure of medication effect in clinical practice can potentially be associated with various problems in individual cases. Notably rating scales rely on the subjective judgment of the rater, thereby risking bias as well as conflicting results. There may be a risk of missing subtle changes in attention, inhibition and activity regulation, which are less evident to an observer and the patients themselves, but that may nevertheless be important to overall function [10]. This in turn may cause a risk of lower adherence and/or giving up medication prematurely due to perceived lack of effect. Low compliance to medication over time is a widespread problem among patients with ADHD [11]. The reasons for this are complex and still not fully understood. There is therefore a need for further research in this area [12].

Continuous performance tests (CPTs) are designed to capture inattention and impulsivity and are therefore often used in the assessment phase of ADHD. Several studies support that they reliably identify ADHD patients and differentiate them from normative controls [13]. However, results are less convincing regarding their ability to differentiate ADHD from other neurodevelopmental disorders in clinically referred samples [14, 15].

QbTest (Qbtech, www.qbtech.com), which is a CPT with an additional motion tracking system, has been used in assessment of ADHD and in treatment follow-up in children and adolescents [16]. Several studies have reported significant improvement in one or more of the parameters captured by the QbTest in response to medical treatment for ADHD in children [17–19]. In an adult population, Bijlenga et al. [10] compared response to stimulant treatment using the QbTest as an objective measure and the ADHD-RS as a subjective measure. They found some significant but rather weak correlations between the two types of measures, including change scores, following medication, which merits further investigation to clarify whether the same correlation exists also in a child population.

Most of the studies mentioned above have been conducted over a fairly limited timeframe, capturing the immediate response to medication. Longer follow-up studies in clinically referred samples using the QbTest as an outcome measure are limited. Looking at one of the conclusions drawn in a review article by Hall et al. [16], further investigation of changes in activity measures when monitoring medication may be of particular interest.

### Objective

The overall objective of this study is two-fold. First, to study patterns of change and stability in the QbTest profiles and their relation to ADHD-RS during one year of ADHD medication, and second, to investigate if QbTest scores or ADHD-RS scores at baseline predict medical compliance. Differences related to gender and diagnosis will also be explored.

## Methods

This study is part of an ongoing single-center open-label 1-year prospective trial of medication for ADHD in children and adolescents, the Clinical Review of QbTest in ADHD (The QbTest trial). The study included patients referred to the Child Neuropsychiatric Clinic (CNC) at Queen Silvia’s Children’s Hospital in Gothenburg, Sweden, from 2014 to 2020. The CNC is an outpatient clinic to which children up until age 18 years are referred for clinical evaluation and neuropsychiatric assessment.

### Procedure

Patients between the ages of 6 and 18 years, with ADHD (any subtype/presentation) according to DSM-5 and with an intellectual ability in the normal range (IQ > 70), according to the Wechsler scales and clinical judgment, were invited to participate in the study. The study protocol includes screening, baseline, and 5 follow-up visits. Investigators in the trial were a pediatrician, a child psychiatrist, and two psychologists, with extensive experience in neuropsychiatric assessment and treatment.

#### Screening

The screening assessment included a review of inclusion and exclusion criteria and was performed by a physician. The exclusion criteria were: (i) physical or psychological limitations making the QbTest unsuitable, (ii) cardiovascular disease, seizures or other unstable medical conditions that might increase the risk for the patient, (iii) bipolar disorder, conduct disorder, psychosis, severe autism or other severe psychiatric or medical conditions making participation unsuitable, (iv) other psychoactive medication, (v) substance use disorder. The screening assessment also included medical, neurodevelopmental and psychiatric evaluation including clinical evaluation of the ADHD diagnosis and subtype/presentation (according to the DSM-5). ADHD symptom severity was measured with the investigator-rated ADHD-Rating Scale-IV [20]. Global functional impairment, quality of life and comorbidity status were evaluated using interviews and questionnaires (these results will be published in detail separately). A medication washout period of one week before baseline for methylphenidate or amphetamine and 2 weeks for atomoxetine was required for participants with an ongoing medical treatment.

#### Baseline

Approximately 2–4 weeks after the screening visit, the baseline assessment was performed by a physician and a clinical psychologist. Inclusion and exclusion criteria were re-assessed and data regarding symptom severity, global functional impairment, vital signs and adverse events were collected by the physician. The overall cognitive ability and adaptive function of the participant was established by the psychologist, either consulting previous medical records or collecting new data through testing with the Wechsler Intelligence Scales (WISC) and The Vineland Adaptive Behavior Scales (VABS), when previous records were older than 2 years or unavailable. The QbTest measuring inattention, impulsivity and hyperactivity was performed. Medication for ADHD (methylphenidate, lisdexamfetamine, atomoxetine or guanfacine as active substances) was initiated or re-initiated on an individual basis regarding medication type and dose, tailored to each participant’s needs.

#### Visit 1

One month after baseline, the first follow-up (visit 1) occurred. QbTest was completed for a second time. In addition, data on symptom severity, global functional impairment, treatment compliance, vital signs and adverse events were collected. This procedure was repeated at visits 2 to 5 (approximately 2, 3, 6 and 12 months after baseline). For some of the patients where the QbTest could not be completed at visit 1, efforts to complete the test at a later visit were made.

#### Visit 5

In addition to the repeated procedure mentioned above, the QbTest and a follow-up assessment of intellectual ability and adaptive functioning were completed by the clinical psychologist at the twelve-month visit, and comorbidity status was assessed by the physician.

The participants continued their medication at all post-baseline visits. Compliance (number of days with missed doses/number of days in period) was measured by parent and patient report, and was generally high (92%, range 72–100%).

The present study includes data from baseline, visit 1 and 5. Data analysis focuses on the results on the QbTest and the ADHD-RS symptom ratings.

### Participants

The first consecutive 95 participants in the QbTest trial were selected for the present study. Fourteen (15%) of these participants declined participation in the trial after screening and three (3%) were excluded due to a diagnosed intellectual disability, leaving 78 participants to be included at baseline, 29 girls (37%) and 49 boys (63%). Mean age at baseline was 12.4 years (SD = 3.6) with an age range of 6–18.1 years. All participants had a confirmed diagnosis of ADHD, *n* = 53 (68%) with combined presentation and *n* = 24 (31%) with inattentive presentation, and one participant (1%) with an unspecified ADHD diagnosis. Twenty participants (26%) also had an autism spectrum disorder (ASD) diagnosis. An additional 15 participants (19%) were described in their medical records to have autistic traits but without meeting criteria for a diagnosis. Other diagnosed neurodevelopmental “comorbid” disorders in the study group were Oppositional-Defiant Disorder (ODD; *n* = 13 (16%), and subclinical ODD in an additional 37 participants (47%), i.e., with several symptoms but not meeting full diagnostic criteria), dyslexia (*n* = 7), developmental coordination disorder (*n* = 7), tic disorder (*n* = 5), and language disorder (*n* = 2). A large majority were treated with stimulants (*n* = 72; methylphenidate or lisdexamfetamine), a few with guanfacine (*n* = 5; of these 3 started with a stimulant at baseline but switched to guanfacine after a few weeks due to adverse effects) or atomoxetine (*n* = 1). The dropout rate after baseline was 18 (dropout related to visit 1 *n* = 4, visit 2 *n* = 2, visit 3 *n* = 3, visit 4 *n* = 4, visit 5 *n* = 5), meaning that 60 of 78 (77%, 22 girls (37%) and 38 boys (63%)) were followed from baseline to visit 5 (12 months after baseline). All but one of those who dropped out of the study were treated with stimulants, the single other patient with guanfacine. Fourteen of the 18 participants who dropped out of the study discontinued their ADHD medication - dropout reasons were lack of efficacy or adverse effects (*n* = 8; stimulant), wished to get by without medication (*n* = 2, stimulant), moved to other area (*n* = 2), other unrelated medical reason (*n* = 2). Four participants continued their medication - dropout reasons were move to other area (*n* = 3), lack of time (*n* = 1). Since they did not continue follow-up in the study, the duration of continued medication is unknown. Non-ADHD medication was limited to Melatonin (*n* = 8) or Hydroxizine (*n* = 2) for sleep, and Sertraline for anxiety/mood (*n* = 2). All patients also received the “standard” psychoeducation to the child, the parents, and the school about the child’s individual needs, which is given to all families after neuropsychiatric assessments at our center. No patients received Cognitive Behavioral Therapy (CBT) or other psychological interventions.

### Instruments

The QbTest [16, 21] is a computer-based CPT, measuring inattention and impulsivity, with an additional motion tracking system designed to measure activity. There are two versions of the test designed for different age groups (QbTest 6–12 and QbTest 12–60) where the task and duration of the test are adjusted to fit the cognitive development of the different age groups. In the first version, the child is presented with a Go/No-Go type task (two types of stimuli; gray circle, defined as target and gray circle with a cross over it, defined as non-target) and the duration time is 15 min. The second version with duration time of 20 min, is based on the unconditional identical pair’s principle, where four types of stimuli (red circle, blue circle, red square and blue square) are randomly presented and the target is defined as the stimuli identical in shape and color to the previous stimuli. The target rate is 50% for QbTest (6–12) and 25% for QbTest (12–60). Twenty-five of the participants were tested with QbTest 6–12, and 48 with QbTest 12–60.

Three cardinal parameters are presented in the test report: QbInattention, QbImpulsivity and QbActivity. These in turn are derived from a total of 10 parameters, four measuring activity (Time Active, Distance, Area, and Micro events), and six measuring impulsivity and inattention (Reaction Time, Reaction Time Variation, Omission Errors, Commission Errors, Normalized Commission Errors and Anticipatory Responses). The subject’s performance is compared to a normative data set of 1307 participants, matching age and gender. The results are presented as age- and gender-specific standardized scores (equal to z-scores) around a normative mean of 0 (standard deviation = 1). Positive scores (> 0) indicate more problems with inattention, impulsivity and hyperactivity, whereas negative scores indicate normal to extremely good performance [21].

The Wechsler intelligence scales [22, 23] are globally the most used test battery to establish general intellectual functioning in children and adults in clinical settings, with adequate psychometric properties. The intelligence scales are individually administered and generate an overall measure of cognitive ability i.e., FSIQ. The mean FSIQ at baseline in the total study group was 90.9 (SD 11.3, range 70–120).

The Vineland Adaptive Behavior Scales (VABS), Second Edition [24] is an instrument designed to capture an individual’s level of adaptive behaviors. VABS generate a total score of the adaptive behavior level as well as scores of domains and subdomains for different adaptive functions. The domains are communication, daily living skills, socialization, motor skills. In the present study, only the baseline total score is reported. The psychometric properties of the Vineland-II have been adequately researched and reported to be robust regarding validity and reliability except for a somewhat lower inter-rater reliability indicating a certain rater effect [24]. The scale is administered either using a semi-structured interview with a parent or a self-administered parent rating scale. Sparrow et al. [24] report that the modes of administration of the scales do not significantly affect the results, with correlations roughly equal to the test–retest reliability (> 0.85) using the same administration method. The interview version was used in 16 cases (21%) and the parent rating scale in 51 cases (65%). The different formats were used due to time restrictions. The interview format takes approx. 1–2 h whereas the rating scale could be completed by the parent at the same time as the participant was assessed cognitively or be completed by the parent at home and sent in afterwards. However, the latter procedure led to failure to collect complete rating scales in 11 cases (14%).

The mean VABS total score at baseline in the total group was 79.4 (SD 15.4, range 42–108), indicating borderline impaired adaptive functioning in the total group but with individual variation ranging from severely impaired to average. No significant score difference was found between those who were interviewed and those who completed the parent rating scale.

The ADHD-RS-IV [20] is an 18-item symptom checklist covering the DSM symptoms of inattention, impulsivity and hyperactivity. The ADHD-RS is scored on a 4-point Likert scale, resulting in a total score and scores on 2 separate subscales: one of inattention and the other of hyperactivity–impulsivity. A higher score reflects greater symptom load. The ADHD-RS is a frequently used instrument in research to evaluate treatment response in children and adolescents with ADHD. The psychometric properties have been investigated and found adequate by the original authors and supported in subsequent independent research [25]. In the present trial, the ADHD-RS was investigator-rated by interview with the parents and children. The total baseline mean on the ADHD-RS was 34.3 in the total group (SD 8.5), indicating moderate to marked ADHD symptom severity.

### Statistical analysis

The statistical software SPSS, version 25, was used for all data analysis. Non-parametric analyses were used due to the relatively small sample size and to reduce the impact of any outliers. The chi-square test for independence was used to calculate whether gender, ADHD subtype, comorbid ASD diagnoses and number of diagnoses were related to dropout. The Mann–Whitney U test was used to compare QbTest results, age, FSIQ and VABS between the drop-out group and remain group. Mann–Whitney U test was also used to check for gender differences in the QbTest results. The Friedman tests and post hoc Wilcoxon signed-rank tests with Bonferroni-adjusted pairwise comparisons were used to calculate the changes in QbTest and ADHD-RS results across the three different time points, before medication, at visit 1 and at visit 5. Finally, to examine individual differences in response to treatment, change (delta) scores were calculated by subtracting the baseline and the visit 1 scores on the QbTest and the ADHD-RS. Spearman correlation analyses were conducted between the change scores to examine whether these indices were associated.

## Results

### The QbTest results across three time points during medication

Fifty-one participants out of the 60 who remained in the study until visit 5 (85%) completed the QbTest across all three time points (Table 1).

**Table 1:** QbTest scores and ADHD-RS scores at baseline, visit 1 and visit 5

	Baseline	Visit 1	Visit 5
Qb scores *N* = 51	50th percentile (*Md*)	50th percentile (*Md*)	50th percentile (*Md*)
QbTotal	1.03	0.47	0.20
QbActivity	1.30	0.60	0.90
QbInattention	1.30	0.70	0.30
QbImpulsivity	0.40	− 0.20	− 0.30
ADHD-RS scores *N* = 60			
ADHD-RS total	35	22	19.5
ADHD-RS attention	19	11	11
ADHD-RS hyp/imp	17	10	9

QB results are expressed in standardized scores around a normative mean of 0 (standard deviation = 1)

A series of non-parametric Friedman signed-rank tests were conducted to examine how scores from the QbTest and ADHD rating scale changed from baseline to visit 1 (after one month) and to visit 5 (after twelve months). Results were consistent in the sense that the effect of time point was significant for all variables (all *Q*-values > 12, *p* values < 0.01). Bonferroni-adjusted pairwise comparisons showed consistently that there were significant reductions in symptoms from baseline compared with visit 1 and visit 5, whereas no significant change was observed between visit 1 and visit 5. No significant differences in the QbTest results related to gender were found.

### Correlations between change scores on the ADHD-RS and QbTest

There was a small, positive correlation between the change (delta) scores on the QbTest total and the change scores on ADHD-RS total (*rho* = 0.28, *n* = 52, *p* < 0.05) from baseline to 1-month follow-up, with improved performance on the QbTest associated with improved ratings on the ADHD rating scales. Regarding sub-scores on the ADHD-RS scale and QbTest, there were moderate significant positive correlations between the QbTest total change score and the ADHD-RS hyperactivity change score. Also, there were moderate significant correlations between the QbTest impulsivity parameter change score and the ADHD-RS total and hyperactivity change scores, respectively. The measures of inattention and of activity change scores on the QbTest did not correlate significantly to any of the other parameters (Table 2). Qb-test and ADHD-RS change scores between baseline and 12-month follow-up and between 1-month and 12-month follow-ups did not correlate significantly.

**Table 2:** Correlations between change scores on the ADHD-RS and QbTest baseline to one month follow-up (Visit 1)

*N* = 52	ADHD-RS total	ADHD-RS attention	ADHD-RS hyperactivity
QbTotal	0.28*	0.08	0.35**
QbActivity	0.24	0.19	0.22
QbInattention	0.07	− 0.03	0.16
QbImpulsivity	0.30*	0.09	0.38**

^**^p < 0.01 (2-tailed)

^*^p < 0.05 (2-tailed)

**Table 3:** General characteristics at baseline of participants who remained in the study and of those who dropped out

	Remained *n* = 60	Dropped out *n* = 18	*p* value
Mean age (SD)	12.5 (3.6)	13.7 (3.9)	0.202
Male *n* (%)	38 (63)	11 (61)	0.864
ADHD inattentive type	17 (28)	7 (39)	0.113
ADHD combined type	43 (72)	10 (56)	
ADHD-unspecified	0 (0)	1 (6)	
Comorbidities			
Autism spectrum disorder	15 (25)	5 (28)	0.482
Autistic traits	12 (20)	3 (17)	
Number of diagnoses			
ADHD only	23 (38)	5 (28)	0.851
ADHD + 1	25 (42)	9 (50)	
ADHD + 2	9 (15)	4 (22)	
ADHD + 3	1 (1.7)	0 (0)	
ADHD + 4	1 (1.7)	0 (0)	
ADHD + 5	1 (1.7)	0 (0)	
Full Scale IQ WISC	91.9 (11.1, *n* = 59)	87.7 (11.5, *n* = 18)	0.166
Vineland-II total score	79.6 (15.9, *n* = 51)	78.7 (13.7, *n* = 12)	0.864

### General characteristics, ADHD-RS scores and QbTest results at baseline for participants who remained in the study and for those who dropped out

No significant difference was found between the group that remained in the study compared to the group that dropped out regarding gender, age, FSIQ, VABS, ADHD presentation, number of diagnoses, or coexisting ASD diagnosis (Table 3).

Comparing the cardinal parameters of the QbTest, the Mann–Whitney *U* test revealed significantly less difficulties on the QbInattention at baseline in the group of participants who dropped out before visit 5 (*Md* = 0.200, *n* = 16), compared to the participants who remained (*Md* = 1.3, n = 57), *U* = 632.5, *z* = 2.36, *p* = 0.019, *r* = 0.28. When extending the analysis to calculate the standard parameters comprising the cardinal parameter of inattention, separately, the parameters measuring reaction time and reaction time variation differed but not the measure of omission errors. The group of dropout participants had both faster reaction time (*Md* = − 0.150, *n* = 16) and lower reaction time variation (*Md* = 0.150, *n* = 16) than the remain group (*Md* = 1.00, *n* = 57) on both measures, respectively (Reaction time *U* = 655, *z* = 2.6, *p* = 0.008, *r* = 0.30 and Reaction time variation *U* = 623.5, *z* = 2.2, *p* = 0.025, *r* = 0.26). No other cardinal parameters or test parameters on the QbTest differed significantly between the drop-out and remain groups (Table 4).

**Table 4:** Comparison between participants who dropped out before visit 5 and participants who remained in the study on QbTest results and ADHD-RS scores at baseline

QbTest means (SD)	Remained *n* = 57^a^	Dropped out *n* = 16^b^	*p* value
QbActivity	1.3 (1.0)	1.2 (0.6)	0.323
QbInattention*	1.2 (1.2)	0.5 (1.2)	0.019*
QbImpulsivity	0.4 (1.3)	0.5 (1.0)	0.645
Inattention			
Reaction time*	0.9 (1.1)	0.1 (0.9)	0.008**
Reaction time var.*	1.1 (1.3)	0.3 (1.2)	0.025*
Omission error	0.9 (0.9)	0.8 (1.2)	0.883
ADHD-RS means (SD)	*n* = 60	*n* = 18	
Total score	34.8 (9.1)	32.4 (5.9)	0.277
Hyper/impulsive	16.1 (6.5)	13.9 (5.1)	0.168
Inattention	18.8 (4.0)	18.6 (3.3)	0.784

^a^Three subjects did not complete the QbTest at baseline

^b^Two subjects did not complete the QbTest at baseline. QB results are expressed in standardized scores around a normative mean of 0 (standard deviation = 1) *p =  < .05, **p = .01

## Discussion

The results of this study indicate that both investigator-rating of ADHD symptoms with the ADHD Rating Scale and a CPT test with motion tracking capture effect of one year of medication. Both measures showed significant and robust reductions in symptoms from baseline to 1 month and 12 months. A correlation between the two measures was found in the total change scores of each instrument, but only in the short term (1 month), and not in all subdomains. Change scores from baseline to 1 month of the ADHD-RS hyperactivity subscale correlated with the QbTotal change score and with QbImpulsivity change score but not with QbActivity change score. We also found that ADHD-RS total change score correlated with QbImpulsivity change score. Bijlenga et al. [10] who similarly investigated the relationship between the ADHD-RS and QbTest in capturing medication effect in an adult population, found the two instruments to correlate moderately on the total change scores and weakly on some subdomains. In our sample of children and adolescents, correlations of the total change scores were slightly weaker but significant. Interestingly, in our child population, correlations between QbImpulsivity and ADHD-RS hyperactivity were the strongest, moderate, while no such correlations were found in the adult population in Bijlenga et al. [10].

Some previous studies have shown that the ability of QbTest to identify ADHD in a clinical sample was moderate, and the ability to differentiate between ADHD subtypes unsatisfactory [14, 15]. Hall et al. [16] reviewed the evidence for the clinical utility of CPT in the assessment phase and subsequent medical titration of ADHD medication. They found that using objective measures of activity was strongly supported by current research in differentiating ADHD from non-ADHD, and these measures appeared to be sensitive to medication effects. In a review by Toplak et al. [26], research on the association between rating scale measures and performance-based tests of executive function was reviewed. Based on 20 studies of which 13 concerned a child population, they found a minority of reported correlations to be significant between the two types of measures and even when significant the correlations were weak. Interpretation of the results was that the different kinds of measures capture different aspects of executive functioning. They reasoned that performance-based measures are highly standardized in administration aiming to achieve optimal performance and thereby capture primarily processing efficiency. Meanwhile, rating scales ask participants to rate typical performance to assess the individual’s goal achievement. In conclusion, the two different kinds of measures are not judged to be interchangeable, but rather provide separate and equally important information in the process of assessment.

Our study findings suggest that the reasoning of Toplak et al. [26] may also be applied to monitoring of ADHD treatment effects with performance-based tests and rating scales of attention, hyperactivity and impulsivity. Based on that interpretation, it makes sense that the QbTest performance and observable symptoms rated in the ADHD-RS measure different aspects of symptoms and executive functions, since only a weak significant correlation between the overall change scores was detected in the short term (1 month), and no significant correlation in the long term (12 months).

Our data support that all the parameters on the QbTest show significant improvement after 1 month and 12 months of medication. We also found some indications that information gained from the QbTest could predict adherence to ADHD treatment. The group who dropped out of the trial, of which a majority stopped medication, had on average less inattentive problems, faster reaction time and less variation in reaction time on the baseline QbTest than those who stayed on medication throughout the study. However, this difference was not seen in the activity parameters or in the impulsivity parameters, nor in the ADHD-RS. No other factors, such as FSIQ, age or gender, differed between the two groups. This might suggest that a CPT test could be a more sensitive indicator about who will continue medication and who will not, since this did not appear to be fully captured in the rating scales. However, to confirm this finding replication is required in a larger sample as well as analyzing subjective reasons for dropping out of the study in more depth.

### Strengths and limitations

The strengths of the present trial are the relatively long follow-up period of 1 year, and the naturalistic study design in which medication was tailored to the needs of each participant, increasing the ecological and clinical validity of the trial. Furthermore, in many studies, cases with ASD are excluded. This was not an exclusion criterion in this trial, which may make the results more applicable to the group of patients with overlapping diagnoses, often seen in clinical practice. In the light of the previous study by Bijlenga et al. [10] in an adult population, our results can possibly extend the validity of those findings to a group of children with more complex neurodevelopmental problems. In addition, several previous studies have used variants of CPT tests in medical follow-up studies measuring attention and impulsivity but with no specific measure of hyperactivity. The CPT test used in the current study measures all three components.

Our study also has important limitations. It is an open-label trial with no control group, which reduces the evidence strength since the longitudinal changes may rely on other factors than medication. Also, the sample size is relatively small. The significant difference found on the inattention parameter of the QbTest between those completing the study and those who dropped out merits further investigation in a larger sample. Another limitation is the heterogeneity of the drop-out group regarding medication status, since 14/18 participants stopped taking medication, whereas 4 continued medication but dropped out of the study.

### Conclusion

Both ADHD-RS and QbTest results appear to capture medication effect, but weak correlations between the two measures suggest that their role in medical follow-up might be complementary rather than interchangeable. Differences in the severity of inattention problems may affect the need and motivation for continued ADHD medication and/or participation in long-term follow-up. Further investigations of the reasons for non-compliance are needed. Results from this study suggest that looking at results from neuropsychological tests including CPT with motion tracking, may shed further light on the cognitive and behavioral profiles of those who benefit from medication and those who do not.

## Data Availability

Study data are available from the authors on reasonable request.
